# A novel dual-capability naphthalimide-based fluorescent probe for Fe^3+^ ion detection and lysosomal tracking in living cells†

**DOI:** 10.1039/d2ra03688f

**Published:** 2022-08-26

**Authors:** Xinran Li, Wenwu Qin

**Affiliations:** Test and Analysis Center, Shenyang Jianzhu University Shenyang 110168 PR China; Key Laboratory of Nonferrous Metal Chemistry and Resources Utilization of Gansu Province and State Key Laboratory of Applied Organic Chemistry, College of Chemistry and Chemical Engineering, Lanzhou University Lanzhou 730000 PR China qinww@lzu.edu.cn +86-931-8912582

## Abstract

We design and synthesize a novel 1,8-naphthalimide-based fluorescent probe MNP that features the dual capabilities of tracking lysosomes in living HeLa cells and sensitively detecting Fe^3+^ ions in aqueous solution. The MNP is obtained by modifying the morpholine group with a lysosomal targeting function and the piperazine group with an Fe^3+^ ion recognition function on the 1,8-naphthalimide matrix. In the presence of Fe^3+^ ions, the MNP acts as a recognition ligand to coordinate with the central Fe^3+^ ion, and the protonated [MNPH]^+^ is eventually generated, in which significant fluorescence enhancements are observed due to the intramolecular photo-induced electron transfer (PET) process being blocked. The limit of detection of Fe^3+^ ions is as low as 65.2 nM. A cell imaging experiment shows that the MNP has low cytotoxicity and excellent lysosomal targeting ability. Therefore, the MNP offers a promising tool for lysosomal tracking and relevant life process research.

## Abstract 2

A newly prepared 1,8-naphthalimide-based fluorescent probe, MNP, allows the detection of Fe^3+^ ions in aqueous medium and lysosomal tracking in living cells. MNP was used *in situ* for the imaging of lysosomes in HeLa cells, a new strategy for lysosome-related medical diagnosis.

## Graphical abstract

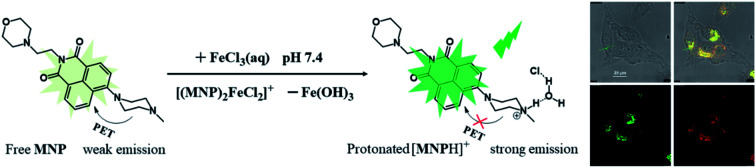

## 1. Introduction

In recent years, subcellular-organelle targeting strategies have evolved significantly and redefined the future development of multifunctional nano drugs and the clinical transformation of precision medicine.^1–3^ As eukaryotic organelles, lysosomes contain many hydrolases and secretory proteins that are active in the pH range of acidic solution (3.5–5.5). Lysosomes are the terminal degradation chambers of living cells, and are involved in many physiological processes such as metabolism, apoptosis, intracellular transport, and immunologic defense. Lysosomal dysfunction can lead to various diseases, especially cancer-related diseases.^4–6^ Therefore, an effective lysosomal tracking strategy for cancer cells is critical for the prevention and treatment of tumor invasion and metastasis, and will be helpful in guiding the diagnosis and treatment of lysosome-related diseases.^7,8^ Compared with other methods, the fluorescent probe method is more ideal for subcellular tracking due to its simplicity, rapid response, good biocompatibility, and high sensitivity.^9,10^ Therefore, it has become an important focus of research to develop novel fluorescent probes that can reversibly monitor lysosomal images to track lysosomes. Recently, a number of fluorescent probes have been reported for lysosomal tracking in living cells. However, there are few reports of dual-capability probe that can track lysosomes and detect the analyte in lysosomes.

Iron is one of the most important trace elements in human body, and it plays a pivotal role in many physiological reactions. There are two main forms of iron in human body, iron storage compounds and iron-containing biologic molecules. Fe^3+^ ions, in particular, play a key part in oxygen transport, oxygen metabolism, transfer of electrons, and many catalytic reactions.^11–14^ The deficiency or overload of iron may lead to biological dysfunction and disturb the cellular homeostasis *in vivo*, which will in turn cause anaemia, diabetes, Alzheimer's disease, liver injury, heart and renal failure, other conditions.^15–20^ Besides, iron ions can also cause environmental pollutions, such as water pollution, which is harmful to human health. As stipulated by the U.S. Environmental Protection Agency, the maximum acceptable content of iron in drinking water should be 5.357 μmol L^−1^.^21^ Therefore, the sensitive and rapid determination of Fe^3+^ ions is essential for the protection of physiological and natural environments. Recent years, a large number of fluorescent probes have been designed and synthesized for the detection of Fe^3+^ ions.^22^ However, Fe^3+^ ions tend to form insoluble Fe(OH)_3_ under aqueous conditions at neutral pH. In lysosomes (pH 3.5–5.5), new iron-containing species such as [Fe(OH)_2_]^+^ and [Fe(OH)]^2+^ can be formed with the release of protons. At present, there are few Fe^3+^ ions fluorescent probes that can detect these ions directly, especially when fluorescence enhancement (turn-on) response is required.^23^

1,8-Naphthalimide and its derivatives play a key role in the fluorescent dye field. They have been extensively applied in biochemistry, polymers, optical storage, fluorescent sensors, and biological medicine due to its good thermal and oxidation stability, high electron affinity, large Stokes shifts, good photostability, and high fluorescence quantum yields.^24–26^ As the “simplest” molecule, 1,8-naphthalimide has better water solubility and an easier functionalization process than other molecules. Moreover, 1,8-naphthalimide is a typical photo-induced electron transfer (PET) dye for fabricating fluorescent probes, which is considered an important strategy for the design of fluorescence sensors. These probes are usually built in the format of “fluorophore-spacer-receptors”.^27^ Based on these characteristics, it can offer ideal fluorophores for fabricating fluorescent sensors. However, few naphthalimide derivative-based sensors are currently available for applications in subcellular imaging.

**Scheme 1:**
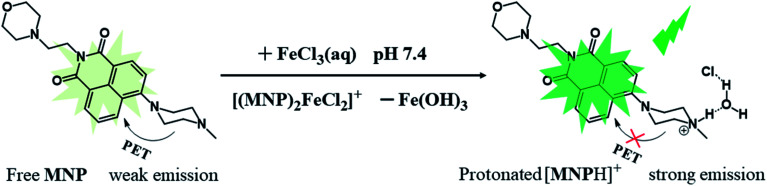
The Concept of MNP for Sensing of Fe^3+^ ions.

In this work, we designed and synthesized a new type of dual-capability fluorescent probe (MNP) based on 1,8-naphthalimide. The probe comes in three parts: (1) a 1,8-naphthalimide group acting as the fluorophore; (2) a morpholine group acting as the lysosomal targeted functional group; (3) a *N*-methyl piperazine group acting as the Fe^3+^ ions recognition group. It has been proved that the MNP can not only obtain lysosomal targeted images of living cells, but also detect Fe^3+^ ions in aqueous solution with high sensitivity. Induced by Fe^3+^ ions, the MNP eventually transforms into protonated [MNPH]^+^ in aqueous solution, which leads to the formation of “turn-on” green fluorescence due to the blocked PET process (Scheme 1). Besides, the applicability of the MNP in bioimaging was examined by confocal fluorescence microscopy. This work provides an innovative idea for the design of dual-capability probes.

## 2. Experimental methods

### 2.1. Materials

All the reagents were of analytical grade and used without further purification. The 2-[4-(2-hydroxyethyl)-1-piperazine]ethanesulfonic acid (HEPES), 4-bromo-1,8-naphthalene anhydride, 2-morpholin-4-ylethanamine, *N*-methyl piperazine, 2-methoxyethanol and triethylamine were purchased from Inno-chem Chemical Co., Ltd (Beijing, China). The Lyso-Tracker Red dye was purchased from Macklin chemical Co., Ltd (Shanghai, China). The methanol, acetone, ethanol, dichloromethane (CH_2_Cl_2_) and metal salts used in the experiments were purchased from Baishi Chemical Co., Ltd (Tianjin, China). The ultrapure water obtained from a Milli-Q ultrapure (18.2 MΩ cm) system was used in all experiments.

### 2.2. Characterization

The ^1^H and ^13^C NMR spectra were obtained with a Bruker DRX-400 spectrometer and CDCl_3_ was used as the solvent. The UV-vis spectra were acquired on a Varian Cary-5000 spectrometer. The corrected steady-state excitation and emission spectra were obtained on an F-7000 spectrometer. Using the time-correlated single photon counting technique in 4096 channels, fluorescence decay histograms were obtained on an Edinburgh Instruments FLS-920 spectrometer equipped with a supercontinuum white laser (400–700 nm). Histograms of the instrument response functions (using a LUDOX scatterer) and sample decays were recorded until they typically reached 1.0 × 10^4^ counts in the peak channel. The quantum yield (QY) of MNP was calculated on an Edinburgh Instruments FLS-920 through a comparative method in which coumarin 6 in methanol was used as the reference. The QY of the MNP was calculated by this formula:
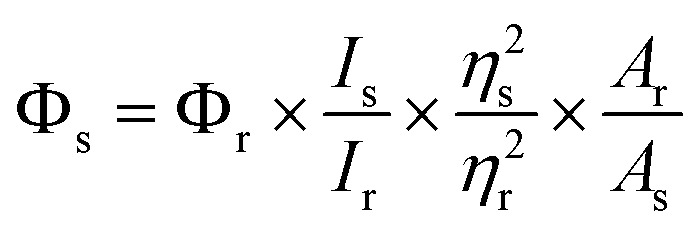
where *Φ* is the QY, *I* is the measured integrated emission intensity, *η* is the refractive index of the solvent, and *A* is the absorption of the reference (r) and as-prepared MNP sample (s). Mass spectra were detected in E.I. mode. High-resolution mass spectra (HR-MS) were obtained on a Bruker micro-TOF-Q II mass spectrometer. Melting points were obtained with an X-4 precise micro melting point cryoscope. To obtain the effects of pH, pH measurements were made with a pH-10C digital pH meter. Cell images were obtained on an FV1000-IX81 confocal laser scanning microscope (Olympus, Japan).

### 2.3. Synthesis of MNP

**Scheme 2:**
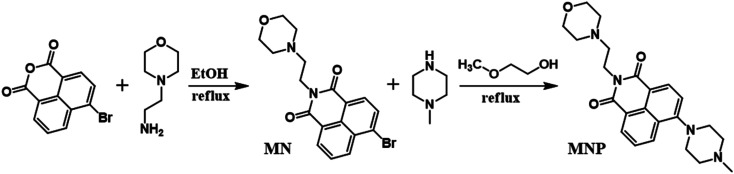
Synthetic route of the MNP.

As shown in Scheme 2, the intermediate product (MN) was synthesized *via* a method similar to that in the literature.^28^ Briefly, 4-bromo-1,8-naphthalene anhydride (1.39 g, 5 mmol) and 2-morpholin-4-ylethanamine (0.67 g, 5 mmol) were added into 30 mL of ethanol. Then the mixture was stirred and refluxed for 4 h. The compound MNP (probe) was synthesized by the following method, which has never been reported before. *N*-Methyl piperazine (0.27 mL, 2.4 mmol) was slowly added dropwise into the MN solution (0.78 g, 1.6 mmol) in 30 mL of 2-methoxyethanol. After 3 mL of triethylamine was added, the mixture was stirred and refluxed for 48 h. After the reaction was complete, the organic solvent was removed and the residue was dissolved in CH_2_Cl_2_ and washed three times (3 × 10 mL) with ultrapure water. After vacuum drying, the crude product was purified by column chromatography on silica gel (CH_2_Cl_2_–MeOH, 50 : 1, v/v) and a yellow solid was obtained. Yield = 68.4%. ^1^H NMR (CDCl_3_): *δ*_H_ 8.57 (d, *J* = 8.0 Hz, 1H, Ha), 8.55 (d, *J* = 8.0 Hz, 1H, Hc), 8.40 (d, *J* = 8.0 Hz, 1H, He), 8.38 (d, *J* = 8.0 Hz,1H), 7.85 (t, 1H), 4.33 (t, 2H, Hf), 3.67 (d, 4H, Hj and Hk), 3.30 (m, 4H, Hl and Hm), 2.74 (m, 4H, Hn and Ho), 2.66 (m, 2H, Hg), 2.58 (m, 4H, Hh and Hi),2.43 (s, 3H, Hp); ^13^C NMR (CDCl_3_): *δ* 37.08, 46.12, 52.92, 53.86, 55.15, 56.25, 67.10, 115.07, 116.70, 123.22, 125.71, 126.18, 129.92, 130.33, 131.13, 132.60, 155.92, 163.98, 164.48. HR-MS calcd for (C_23_H_28_N_4_O_3_) [M + H]^+^ 409.2161, found 409.3139 (Fig. S8†).

### 2.4. Detection of Fe^3+^ ions

The detection of Fe^3+^ ions was performed at room temperature in HEPES buffer. The aqueous solutions of Fe^3+^ ions and other metal ions with different concentrations were freshly prepared before use, and some of them were stored in acidic conditions. To evaluate the sensitivity towards Fe^3+^ ions, different concentrations of Fe^3+^ ions were added into the HEPES buffer containing 10 μM MNP, and equilibrated for 5 min before spectral measurements. The fluorescence spectra were recorded by F-7000 spectrometer with an excitation wavelength of 405 nm.

The river water sample was obtained from Hunhe River of Shenyang, Liaoning Province, China. The sample was first centrifuged at 8000 rpm for 10 min to remove main impurities and then filtered with 0.22 μm membrane. The river water samples with various concentrations of Fe^3+^ ions were added to the MNP sensing system and then the fluorescence spectra were collected.

### 2.5. Cell culture

To obtain the cell permeability of the MNP, HeLa cells were cultured in Dulbecco's Modified Eagle Medium (DMEM) subjoined with 10% (v/v) fetal bovine serum (FBS). The cell lines were maintained under a humidified atmosphere of 5% CO_2_ and at 37 °C. HeLa cells were treated with 10 μM MNP in 1.0 mL of fresh culture medium for 30 min and then treated with 100 μM Fe^3+^ ions for another 30 min, which were compared with those in the blank experiment. Before the cell imaging experiment, HeLa cells were washed three times with PBS buffer to remove free compounds. Confocal fluorescence images of HeLa cells were captured on an Olympus FV1000-IX81 laser confocal microscope.

Moreover, a standard 3-(4,5-dimethylthiazol-2-yl)-2,5-diphenyltetrazolium bromide (MTT) assay was performed to evaluate the cytotoxic effect of the MNP. HeLa cells were seeded in 96-well assay plates at a density of 10^4^ cells per well (100 μL total volume/well) for 24 h. Then, various concentrations of MNP (10, 20, 40, 60, 80 and 100 μM) were added to the serum-free medium and incubated with HeLa cells for 24 h. The optical absorbance of the cells was detected through a microplate reader (German Berthold Mithras2LB943) at 450 nm. The assay was performed in five sets for each concentration of MNP and the control experiment was conducted by measuring the growth culture medium without the MNP.

## 3. Results and discussion

### 3.1. Synthesis and characterization

**Scheme 3:**
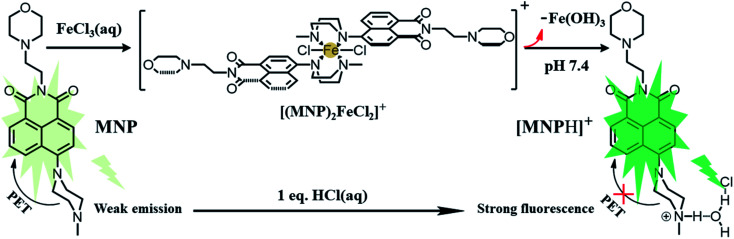
A plausible mechanism of the MNP for ultrasensitive sensing of Fe^3+^ ions.

In this work, the probe (MNP) was designed using 1,8-naphthalimide as the fluorophore, which is a typical PET-based fluorescent dye. The MNP can be synthesized by the intermediate product MN and *N*-methyl piperazine in 2-methoxyethanol, with the -Br group in the MN substituted by the piperazine group (Scheme 2). In the MNP molecules, the 1,8-naphthalimide group acted as the electron acceptor and the piperazine group as the electron donor, forming an electron transfer system within the molecules and inducing a PET process under the excitation of light. The active PET process also “turned off” the fluorescent signal of the MNP, so that the MNP had very weak fluorescence. A protonated species [MNPH]^+^ with strong fluorescence would be obtained when the MNP was treated with either excessive FeCl_3_ aqueous solution or HCl (Scheme 1). It has been reported that the coordination compounds of FeCl_3_ with various modified *N*-aryl or *N*-alkylpiperazine ligands. When Fe^3+^ ions act as the central metal ions to coordinate with these ligands, the ligands may act as a bidentate ligand for coordination, which adopts a boat configuration with a stoichiometric ratio of 2 : 1.^29^ The Job's plot analysis also showed that the binding mode of MNP ligands to Fe^3+^ ions was 2 : 1 (Fig. S1†). However, the rapidly formed intermediate species [(MNP)_2_FeCl_2_]^+^ cannot be extracted at laboratory scale from wet solvents used for fluorescence sensing and bioimaging experiments (Fig. S3†). This was because it rapidly decomposed to form a species [MNPH]Cl, which blocked the PET process in the system and significantly enhanced fluorescence (PET inactive) to achieve the fluorescence sensing of Fe^3+^ ions (Scheme 3). These new compounds were characterized by ^1^H and ^13^C NMR spectroscopies and HR-MS (Fig. S5–S10†).

**Fig. 1:**
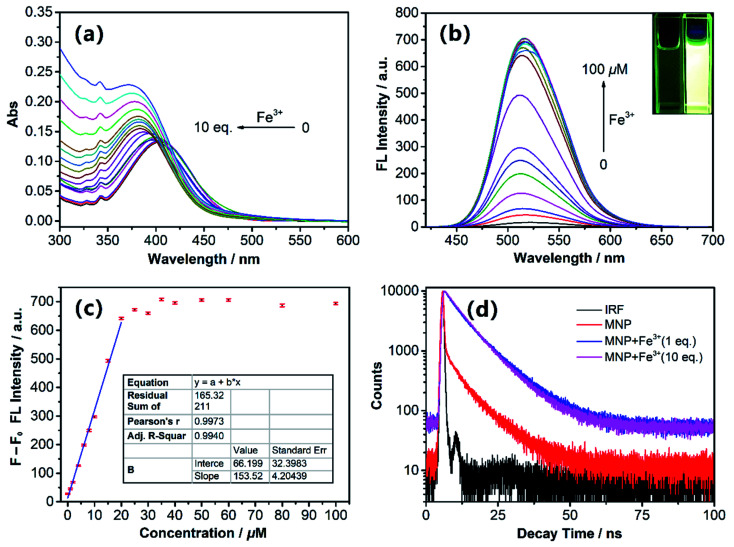
(a) The UV-Vis absorption spectra and (b) fluorescence spectra of 10 μM MNP in the presence of various concentrations of Fe^3+^ ions (0–100 μM) in 10 mM HEPES buffer (pH 7.4; EtOH : H_2_O = 5%; v/v). Inset: photograph of the MNP with the concentration of Fe^3+^ ions at 0 and 100 μM under a UV lamp. (c) Linear fitting curve of relative fluorescence intensity *versus* different concentrations of Fe^3+^ ions. *F*_0_ represents the fluorescence intensity of the MNP without Fe^3+^ ions, and *F* represents the fluorescence intensity with different concentrations of Fe^3+^ ions. (d) Fluorescence decay profiles of the MNP with Fe^3+^ ions in HEPES buffer.

We prepared the stock solution of the free MNP in EtOH, and then investigated the UV-vis absorption and fluorescence response to Fe^3+^ ions at room temperature in 10 mM HEPES buffer (pH 7.4; EtOH : H_2_O = 5%; v/v). Under this condition, the free MNP exhibited one main absorption band centred at ∼405 nm, which can be attributed to a π–π* transition. After the addition of Fe^3+^ ions (0–10 eq. FeCl_3_) into 10 μM MNP solution in the above-mentioned 10 mM HEPES buffer, the maximum absorption peak intensity increased gradually and exhibited a distinct blue-shift from 405 to 372 nm (Fig. 1a). Fig. 1b shows the fluorescence changes of the MNP in the absence and presence of Fe^3+^ ions in HEPES buffer (pH 7.4). When excited with an optimal excitation wavelength of 405 nm, the free MNP only showed weak fluorescence emission (black line) at ∼510 nm due to the active PET process. However, when the concentration of the Fe^3+^ ions increased from 0 to 100 μM, the intensity of fluorescence emission increased significantly at ∼510 nm, which can be attributed to the initial capture of Fe^3+^ ions by the MNP molecules. This resulted in a lower electron-donating ability of piperazine-nitrogen, thus blocking the PET process. Besides, the protonated species [MNPH]^+^ was finally generated in a very short time, which led to the recovery of strong fluorescence emission (Scheme 3). Furthermore, the data analysis exhibited an excellent linear relationship (*R* = 0.997) between the relative fluorescence intensity (*F* − *F*_0_) at ∼510 nm and the concentration of Fe^3+^ ions (0–20 μM). Meanwhile, the limit of detection (LOD) was 65.2 nM, indicating that the MNP is a highly sensitive fluorescent probe (Fig. 1c). The LOD was obtained by the 3*σ*/*k* method, where *σ* is the standard deviation of the blank sample and *k* is the slope of the calibration curve.^30^ The quantum yield *Φ*_s_ of the MNP was 0.01 ± 0.004, and in the presence of Fe^3+^ ions (100 μM), it increased to 0.272 ± 0.008 due to the PET process.

Fluorescence decay traces of the MNP with Fe^3+^ ions were recorded at 510 nm by the single-photon timing method. In the presence of Fe^3+^ ions (1 eq. and 10 eq.), the fluorescence decay can be fitted to the double-exponential profile with lifetimes of ∼3.55 ns, ∼9.4 ns and 4.61 ns, 8.92 ns in HEPES buffer; the average fluorescence lifetimes were 8.77 ns and 8.11 ns, respectively. As shown in Fig. 1d, after the addition of Fe^3+^ ions, the MNP fluorescence decay became slower and the average life time became longer significantly. These results can be attributed to the fact that the PET process was blocked by the reaction with Fe^3+^ ions.

### 3.2. Selectivity to M^3+^ metal cation

**Fig. 2:**
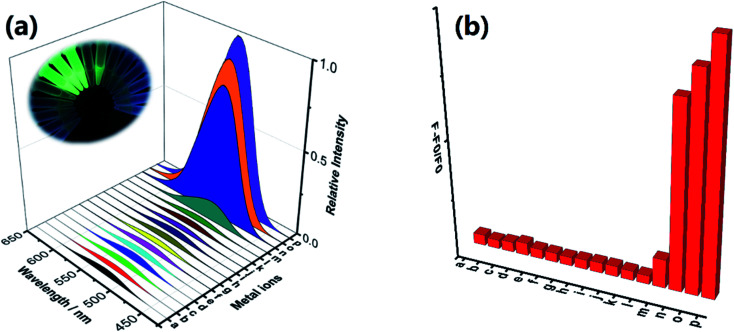
(a) Fluorescence response of the MNP in the presence of various metal ions. The concentration of metal ions was 10 eq. of the MNP in HEPES buffer. Inset: fluorescence changes excited by a UV lamp in the MNP upon addition of various metal ions. (b) The relative fluorescence intensity changes of the MNP in the presence of metal ions (a: MNP, b: Ca^2+^, c: Hg^2+^, d: Co^2+^, e: K^+^, f: Mn^2+^, g: Na^+^, h: Ni^2+^, i: Pb^2+^, j: Fe^2+^, k: Mg^2+^, l: Ag^+^, m: Cu^2+^, n: Cr^3+^, o: Al^3+^, p: Fe^3+^).

In addition to good sensitivity, good specificity was also required. The selectivity of the MNP to Lewis acids such as Fe^3+^, Cr^3+^ and Al^3+^ ions was evaluated by screening its fluorescent response to various biological ions and toxic metal ions in HEPES buffer (pH 7.4; EtOH : H_2_O = 5%; v/v). As shown in Fig. 2, under the same condition, the addition of trivalent cation such as Fe^3+^, Cr^3+^ and Al^3+^ ions resulted in a significant fluorescence enhancement, and no obvious changes in fluorescent signal were observed by adding 10 eq. of various biological ions (Ca^2+^, Co^2+^, K^+^, Mn^2+^, Na^+^, Ni^2+^, Fe^2+^, Mg^2+^ and Cu^2+^) and toxic metal ions (Hg^2+^, Pd^2+^ and Ag^+^). The obtained results demonstrated that the probe MNP has high selectivity and sensitivity towards trivalent cation Lewis acids such as Fe^3+^, Cr^3+^ and Al^3+^ ions, especially aqueous Fe^3+^ ions, which are essential for living organisms and are abundantly present on Earth. To demonstrate the application of the MNP in complicated environment, we further detected the concentration of Fe^3+^ ions in tap water and river water samples. As shown in Tables S1 and S2† recoveries of different known amounts of added Fe^3+^ ions were obtained from 98.4% to 113.5% in tap water samples and 96.7% to 109.3% in river water samples. Therefore, the probe can be applied to the sensing of trivalent cations and the rapid detection of Fe^3+^ ions with high sensitivity and selectivity under specific conditions.

### 3.3. Photostability and pH dependence

**Fig. 3:**
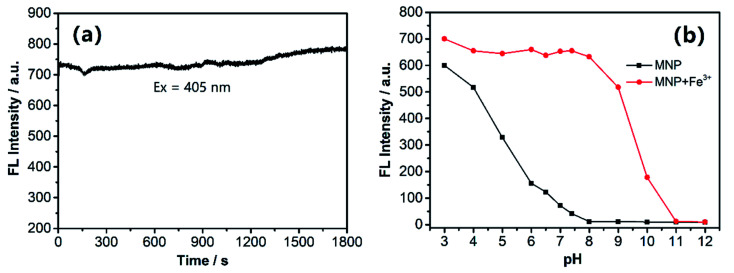
(a) Fluorescence intensity change of the MNP during continuous excitation with a UV beam (ex = 405 nm). (b) Fluorescence intensity of the MNP in the absence and presence (10 eq.) of Fe^3+^ ions as a function of pH values.

We investigated the photostability of the MNP in HEPES buffer (pH 7.4; EtOH : H_2_O = 5%; v/v) and as shown in Fig. 3a, the results revealed that the MNP has excellent photostability and its fluorescence emission intensity at ∼510 nm remained almost unchanged under continuous illumination with a UV lamp for 30 min at room temperature. In addition, we investigated the fluorescence responses of the MNP to Fe^3+^ ions in HEPES buffer (EtOH : H_2_O = 5%; v/v) at various pH values. Furthermore, the fluorescence intensity changes of MNP were independently tested in a wide range of pH (3–12). With the increase of pH values, the MNP showed a quenching trend, the fluorescence intensity was almost entirely quenched at pH > 8. Under acidic condition, the “turn-on” fluorescent signal was observed, which is very suitable for fluorescence imaging of lysosomes. The fluorescence intensity of the MNP in the presence of (10 eq.) Fe^3+^ ions was almost constant within a pH range of 3–8, and the fluorescence quenching of MNP + Fe^3+^ started at approximately pH 8 and entirely quenched at pH > 11. Therefore, we could conclude that the MNP has the potential to be used as a sensitive “turn-on” fluorescent probe for detecting Fe^3+^ ions in the biologically relevant pH range *in vitro* (Fig. 3b).

### 3.4. Cellular imaging

For the applicability of the MNP in living cell imaging, its cytotoxicity is a significant consideration. The cytotoxicity of the MNP was evaluated by the conventional MTT assay in living HeLa cells. After treatment with the MNP at a concentration of 100 μM at 37 °C for 24 h, the cell viability of HeLa showed no significant decrease (Fig. S4†), which indicated that the MNP prepared in this study has good biocompatibility and low cytotoxicity in living cells. Consequently, it can be predicted that the MNP is effective in tracking lysosomes in living cells.

**Fig. 4:**
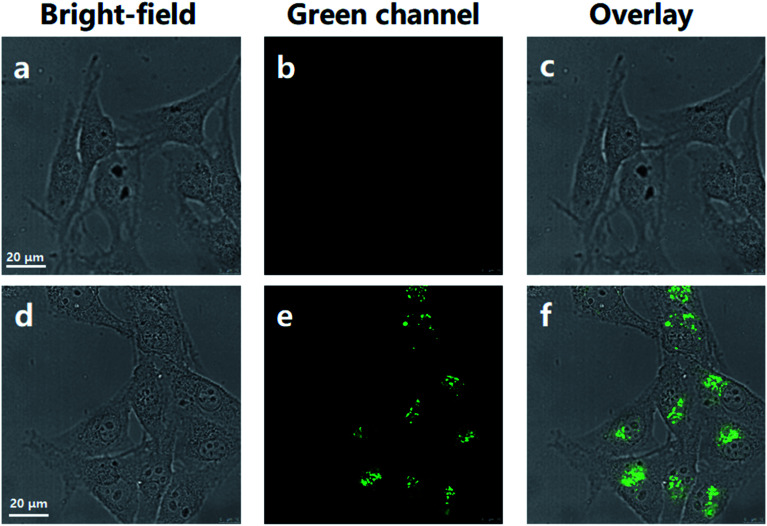
Confocal fluorescence microscopy imaging of HeLa cells. (a–c) Normal HeLa cells. (d–f) HeLa cells cultured with MNP (10 μM) and 10 eq. Fe^3+^ ions for 30 min. Scale bar = 20 μm.

Based on the above results, we assessed the applicability of the MNP in the fluorescence imaging of lysosomes *in vitro*. As shown in Fig. 4a–c, the imaging of normal HeLa cells under the confocal fluorescence microscope has no fluorescence (blank experiment). Unlike the blank experiment, HeLa cells were incubated with 10 μM MNP and 10 eq. Fe^3+^ ions at 37 °C for 30 min, and then the results were analyzed by confocal fluorescence microscopy. A significant unevenly distributed punctate green fluorescence image was observed in the living HeLa cells (Fig. 4d–f). The experimental results revealed that the MNP has good membrane permeability and can be used for fluorescence imaging in living cells.

**Fig. 5:**
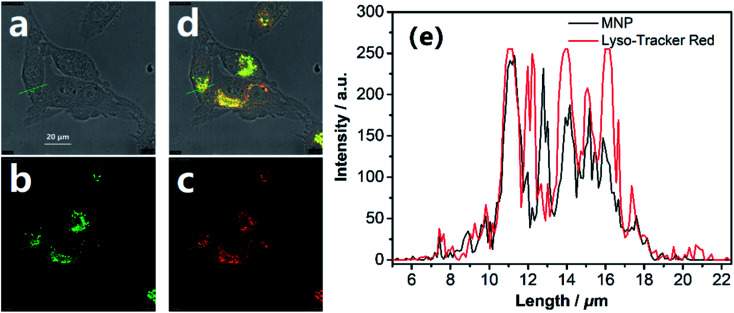
The lysosomal tracking ability and the precise location of the MNP in lysosomes. Confocal fluorescence images of (a) bright-field; (b) the MNP (green) and (c) Lyso-Tracker Red (red, lysosomal red fluorescent probe). (d) Merged image of (a–c). (e) Intensity plot of the MNP and Lyso-Tracker Red in region of interest. Scale bar = 20 μm.

To examine the suitability of the MNP for *in situ* imaging of lysosomes, co-localization tests were performed by co-staining HeLa cells with a commercial Lyso-Tracker Red, a living cell lysosomes tracker along with the MNP. Confocal fluorescence images of the MNP with Lyso-Tracker Red were recorded in separate optical inspection windows, with minimum interference between each other. The MNP showed punctate fluorescence extremely similar to Lyso-Tracker Red under the confocal fluorescence microscope, and a large area of overlap appeared in the fluorescence overlay image (Fig. 5a–d). The intensity curve analysis of the region of interest also showed that the peak location and peak intensity of the MNP are basically identical with that of the Lyso-Tracker Red (Fig. 4e). The above co-localization test results demonstrated that the MNP has excellent lysosomal targeted imaging capability and the potential to be applied for tracking lysosomes in living cells.

## 4. Conclusions

In summary, we designed a novel 1,8-naphthalimide-based fluorescent probe MNP with dual capabilities for rapid and sensitive detection of Fe^3+^ ions *in vitro* and for *in situ* imaging of lysosomes in living HeLa cells. When the piperazine group in MNP molecules are bound to Fe^3+^ ions as recognition ligands, it can block the PET process and restore the strong fluorescence emission, which offers high sensitivity to Fe^3+^ ions in the aqueous medium, and the LOD is 65.2 nM. In the actual sample analysis, the good recoveries ranging from 96.7% to 113.5% also illustrated that the application of the MNP in biological and food sample analysis was anticipated to be promising. In addition, due to its excellent lysosomal targeted imaging ability confirmed by co-localization tests, the probe MNP can also be used to monitor the morphological changes of lysosomes in living cells in real time, which provides a new strategy for lysosome-related medical research and clinical diagnosis. Thus, we believe that this simple and cost-effective dual-capability probe strategy will find wide applications in biochemical and medical fields.

## Conflicts of interest

There are no conflicts to declare.

## Supplementary Material

RA-012-D2RA03688F-s001
