# Supplementary material for: A novel dual-capability naphthalimide-based fluorescent probe for Fe3+ ion detection and lysosomal tracking in living cells
Source: RSC Adv. 2022 Aug 26;12(37):24252–9. doi: 10.1039/d2ra03688f (PMC9413951; doi:10.1039/d2ra03688f)
Supplement: RA-012-D2RA03688F-s001 [file RA-012-D2RA03688F-s001.pdf]

Electronic Supplementary Material (ESI) for RSC Advances.
This journal is © The Royal Society of Chemistry 2022

Electronic Supplementary Information (ESI)

A novel dual-capability naphthalimide based fluorescent probe for Fe^{3+} ions detection and lysosomal tracking in living cells

*Xinran Li,[a] Wenwu Qin.[b]**

[a] *Test and Analysis Center, Shenyang Jianzhu University, Shenyang, 110168, People's Republic of China.*

[b] *Key Laboratory of Nonferrous Metal Chemistry and Resources Utilization of Gansu Province and State Key Laboratory of Applied Organic Chemistry, College of Chemistry and Chemical Engineering, Lanzhou University, Lanzhou 730000, People's Republic of China.*

* Corresponding author: Fax: +86-931-8912582 E-mail address: qinww@lzu.edu.cn (W. Qin).

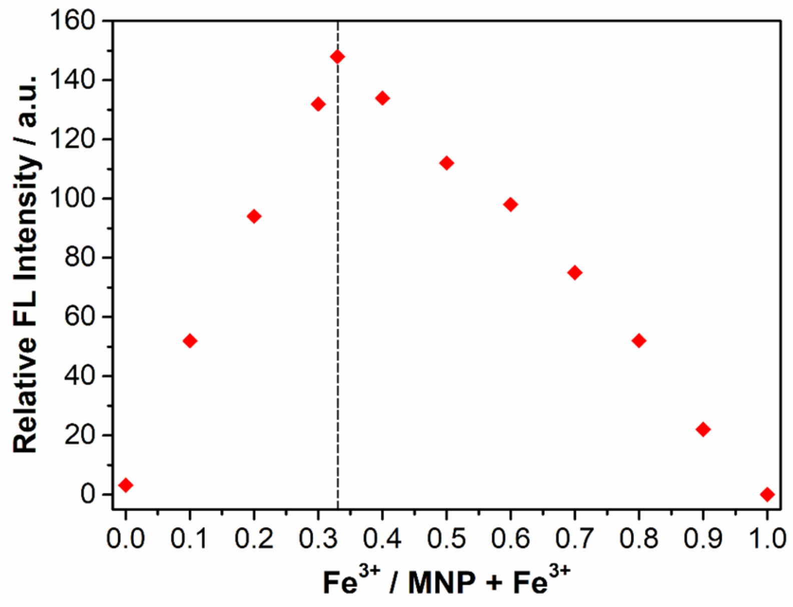

Figure S1 Job's plot of probe **MNP** in 10 mM HEPES buffer (pH 7.4; EtOH: H_2O = 5%; v/v). The total concentration of probe **MNP** and Fe^{3+} ions is 100 μM.

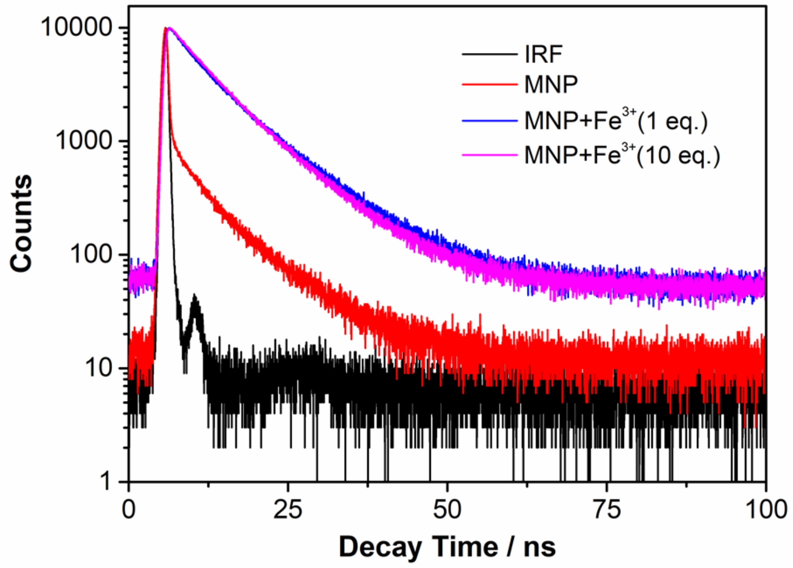

Figure S2 Fluorescence decay profiles of the **MNP** with Fe^{3+} ions. The average lifetime of the **MNP** is too short to fit. The average lifetime of the **MNP** with 1 eq. Fe^{3+} ions is 8.77 ns and contains two lifetime components: 3.55 ns (~24.2%) and 9.40 ns (~75.8%). The average lifetime of probe **MNP** with 10 eq. Fe^{3+} ions is 8.11 ns and contains two lifetime components: 4.61 ns (~30.97%) and 8.92 ns (~69.03%) (delay time at 510 nm emission). The fluorescence decay curves determined at the excitation of 405 nm, and the average lifetime was calculated

according to $\bar{\tau} = \frac{\sum_{i=1}^{n} \alpha_i \tau_i^2}{\sum_{i=1}^{n} \alpha_i \tau_i}$.

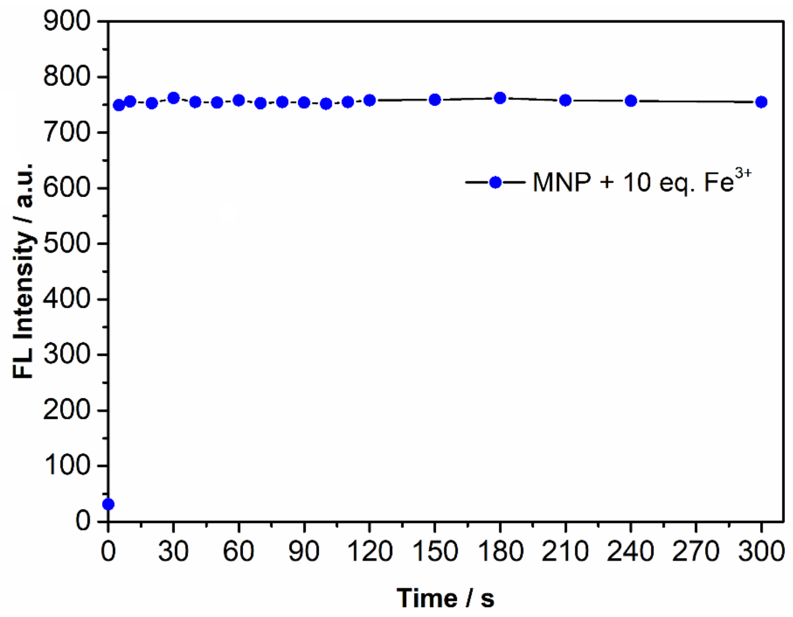

Figure S3 Time-dependent fluorescence intensity changes curve of the **MNP** (10 μM) in the present of 100 μM Fe^{3+} ions in 10 mM HEPES buffer (pH 7.4; EtOH: H_2O = 5%; v/v) (ex = 405 nm).

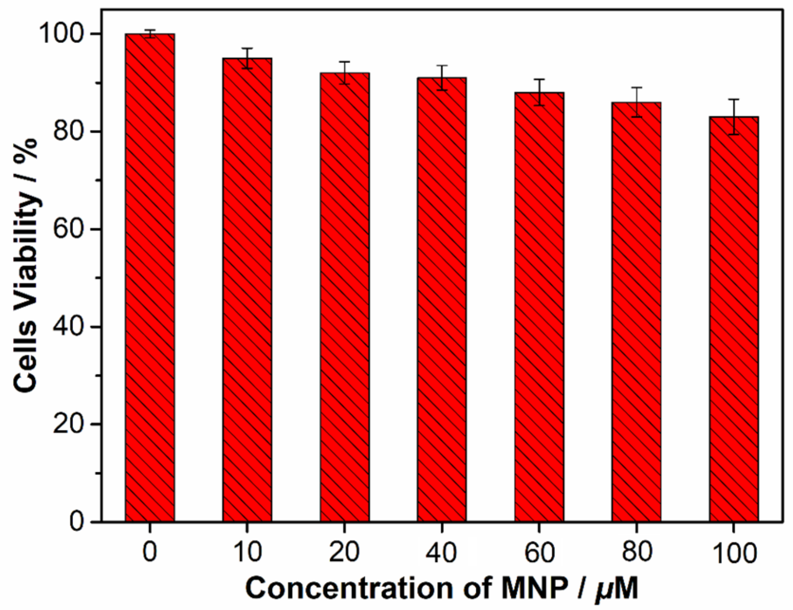

Figure S4 Effects of the **MNP** at varied concentrations on the viability of Hela cells. The cell viability data were checked five times.

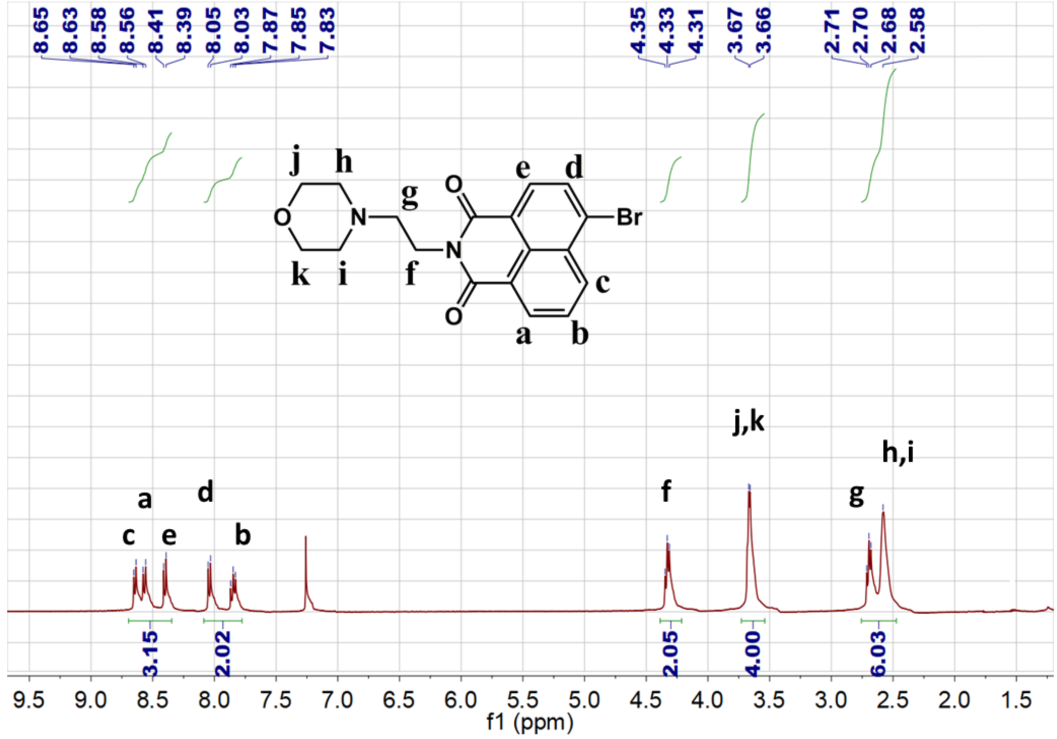

Figure S5 ^{1}H NMR ($CDCl_3$, 400 MHz) spectrum of the **MN**.

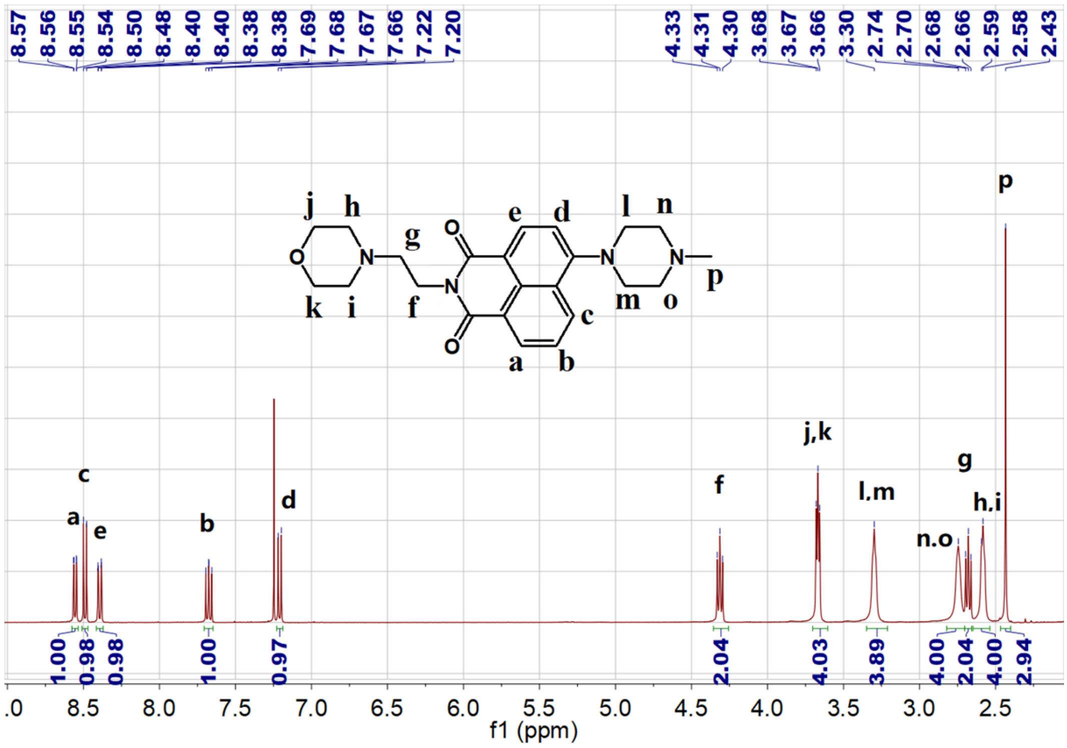

Figure S6 ^{1}H NMR ($CDCl_3$, 400 MHz) spectrum of the **MNP**.

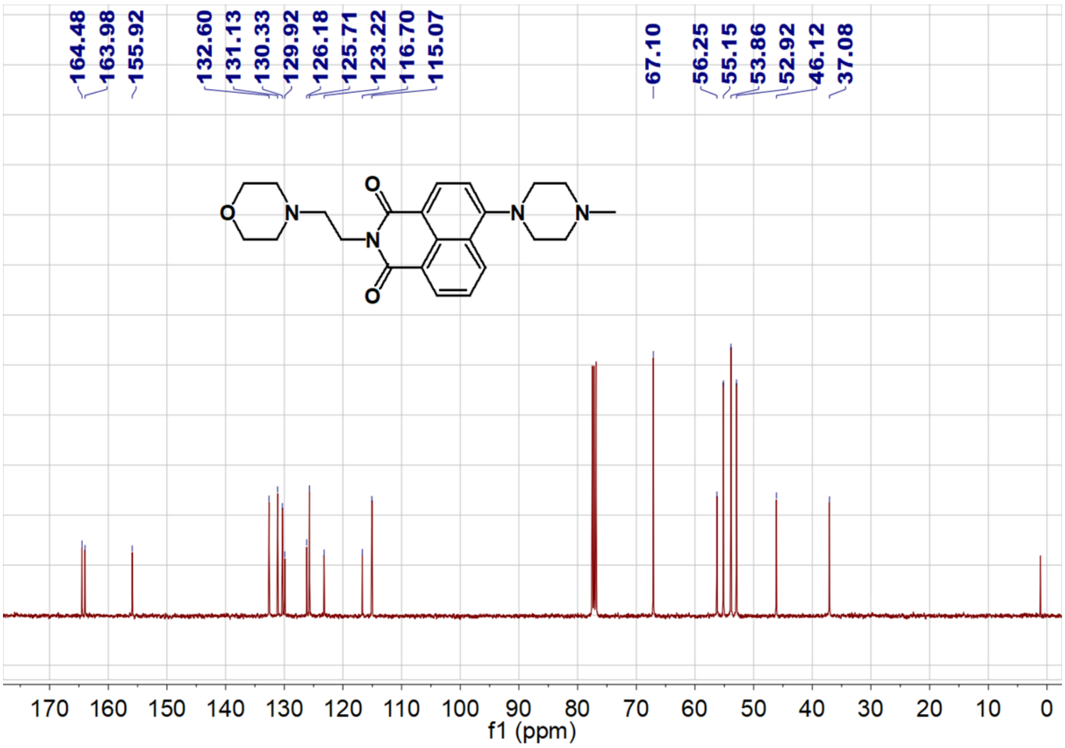

Figure S7 ^{13}C NMR ($CDCl_3$, 400 MHz) spectrum of the **MNP**.

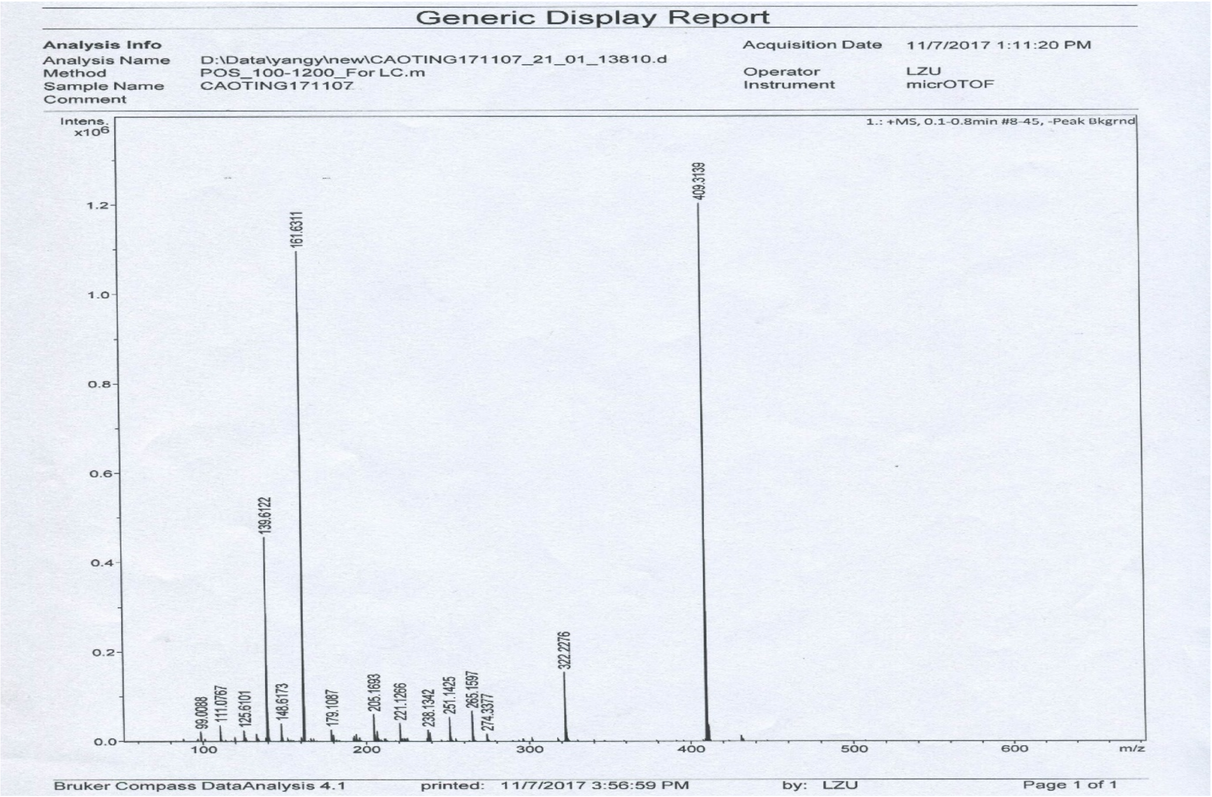

Figure S8 MS Spectrum of the **MNP**, calcd for ($C_{23}H_{28}N_4O_3$) $[M+H]^+$ 409.2161, found 409.3139.

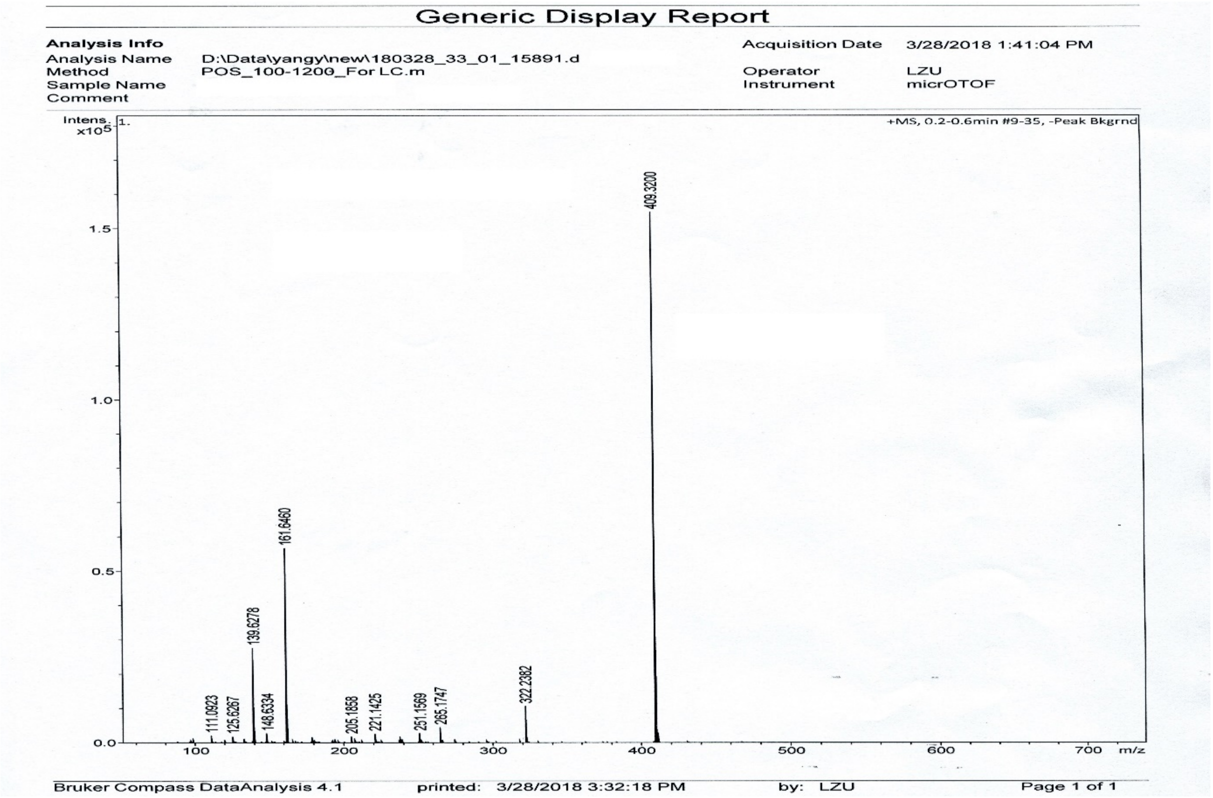

Figure S9 MS Spectrum of the **MNP**+HCl (1 eq.).

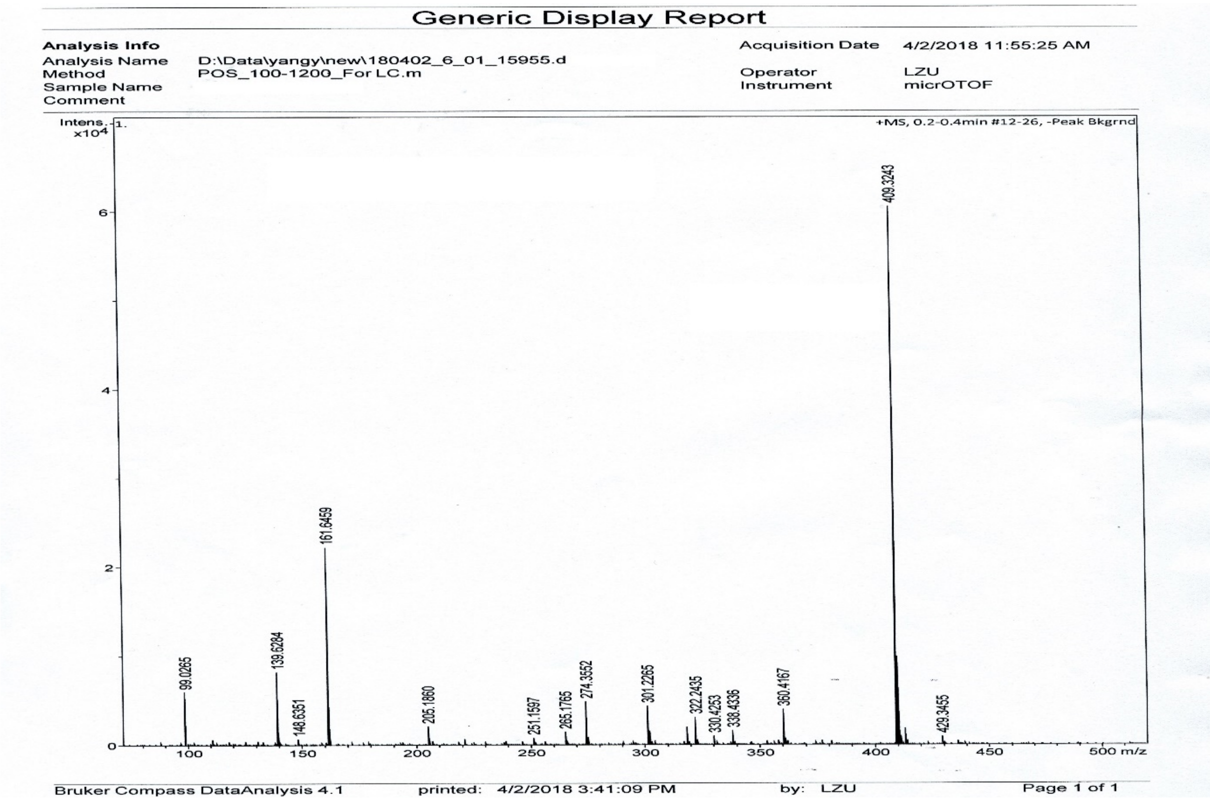

Figure S10 MS Spectrum of the **MNP**+Fe^{3+} (10 eq.).

Table S1 Determination results of the **MNP** in tap water samples.

**Added / μM**	**Detection / μM**	**Recovery / %**	**RSD / n = 3, %**
2	2.27 ± 0.29	113.5	0.52
4	4.30 ± 0.26	107.6	0.57
8	8.38 ± 0.34	104.8	0.37
12	13.31 ± 0.32	101.9	0.42
20	19.68 ± 0.38	98.4	0.44

Table S2 Determination results of the **MNP** in river water samples.

**Added / μM**	**Detection / μM**	**Recovery / %**	**RSD / n = 3, %**
2	2.19 ± 0.33	109.3	0.51
4	4.23 ± 0.29	106.4	0.49
8	8.22 ± 0.35	102.7	0.42
12	11.87 ± 0.36	98.9	0.38
20	19.34 ± 0.47	96.7	0.46
